# Association between prognostic nutritional index and prognosis in acute graft-versus-host disease following allogeneic hematopoietic stem cell transplantation: a retrospective cohort study

**DOI:** 10.3389/fnut.2025.1661993

**Published:** 2025-11-28

**Authors:** Gangping Li, Di Zhang, Yongqi Wang, Fangfang Yuan, Minghui Li, Yuewen Fu

**Affiliations:** 1 Department of Hematology, The Affiliated Cancer Hospital of Zhengzhou University & Henan Cancer Hospital, Zhengzhou, China; 2 Department of Medical Records Management, The Affiliated Cancer Hospital of Zhengzhou University & Henan Cancer Hospital, Zhengzhou, China

**Keywords:** prognostic nutritional index, acute graft-versus-host disease, allogeneic hematopoietic stem cell transplantation, prognosis, association

## Abstract

**Background:**

Evidence on the association between prognostic nutritional index (PNI) and prognosis in acute graft-versus-host disease (aGVHD) following allogeneic hematopoietic stem cell transplantation (allo-HSCT) is limited.

**Objective:**

This study aims to investigate the relationship between PNI levels and prognosis in patients experiencing aGVHD following allo-HSCT.

**Methods:**

We conducted a retrospective cohort study of patients who underwent allo-HSCT at Henan Cancer Hospital from January 2019 to December 2024. Eligible patients were those diagnosed with aGVHD following allo-HSCT. Data on PNI levels, clinical outcomes, and demographics were extracted from medical records. The primary outcomes were overall survival (OS) and event-free survival (EFS). Multivariable Cox regression and subgroup analyses were performed to assess the relationship between PNI levels and prognosis, adjusting for confounders.

**Results:**

This study included 109 eligible patients with a mean age of 29.4 ± 15.3 years. Over a 30-month follow-up, there were 69 deaths, 3 relapses/progressions, and 37 survivors. Among the participants, 51 had Grade I-II aGVHD, and 58 had Grade III-IV aGVHD. After adjusting for confounders, the adjusted hazard ratios (HR) for PNI and OS in T2 (35.8–42.5) and T3 (42.5–59.7) were 0.52 (95% CI: 0.23–0.86, *p* = 0.049) and 0.43 (95% CI: 0.18–0.73, *p* = 0.013), respectively, compared to individuals with lower T1 (20.2–35.8). For EFS, the adjusted HR values for PNI in T2 (35.8–42.5) and T3 (42.5–59.7) were 0.32 (95% CI: 0.17–0.59, *p* = 0.026) and 0.31 (95% CI: 0.16–0.56, *p* = 0.021), respectively, when compared to those with lower T1 (20.2–35.8). These results suggest a potential association between lower PNI levels and poorer prognosis. Kaplan–Meier analysis demonstrated poorer OS (*p* = 0.047) and EFS (*p* = 0.078) in the lower PNI group. Subgroup and interaction analyses revealed no significant interactions by age, sex, CD34+ count and ABO match (all *p* > 0.05), confirming the stability of the association. Sensitivity analyses further supported this consistent association.

**Conclusion:**

Our study underscores the association between lower PNI levels and poorer prognosis in aGVHD patients following allo-HSCT, emphasizing the need for further research to validate PNI as a reliable prognostic biomarker.

## Introduction

Acute graft-versus-host disease (aGVHD) remains a life-threatening complication following allogeneic hematopoietic stem cell transplantation (allo-HSCT), occurring in 30–70% of recipients and contributing significantly to non-relapse mortality (NRM) despite advancements in prophylaxis and treatment strategies (1–3). The pathophysiology of aGVHD involves donor-derived immune cells attacking host tissues, particularly the skin, liver, and gastrointestinal tract, driven by dysregulated inflammatory responses and impaired immune tolerance (4). While corticosteroids remain the cornerstone of first-line therapy, approximately 40–60% of patients exhibit steroid resistance, necessitating second-line interventions with variable efficacy and substantial toxicity (5, 6). This underscores the urgent need for reliable prognostic biomarkers to guide risk stratification and personalized therapeutic approaches.

In recent years, the interplay between nutritional status, systemic inflammation, and immune dysregulation has emerged as a critical determinant of outcomes in allo-HSCT recipients (7, 8). Malnutrition, characterized by hypoalbuminemia and lymphopenia, exacerbates tissue damage and impairs mucosal barrier integrity, fostering a pro-inflammatory microenvironment conducive to aGVHD progression (9). The prognostic nutritional index (PNI), calculated as serum albumin (g/L) + 5 × lymphocyte count (×10^9^/L), integrates both nutritional and immunological parameters, offering a simple yet robust tool to assess systemic resilience (10–14). Previous studies have validated PNI as a predictor of survival in critically ill and geriatric oncology populations, where low PNI correlates with increased infection risks, prolonged hospitalization, and mortality (15–17). However, its prognostic utility in the context of aGVHD—a condition marked by hypermetabolic stress and immune exhaustion—remains underexplored.

Current research gaps persist in understanding how pre-transplant nutritional status modulates post-transplant immune reconstitution and aGVHD severity (8). Although microbiome diversity and short-chain fatty acid (SCFA) production, such as butyrate, have been linked to improved survival and reduced aGVHD incidence through gut barrier stabilization and regulatory T-cell induction (18, 19), the role of systemic nutritional metrics like PNI in this interplay is unclear. Furthermore, while novel therapies targeting tissue repair (e.g., GLP-2 analogs) or cytokine pathways (e.g., JAK inhibitors) show promise in refractory GVHD (7), their efficacy may depend on baseline patient nutritional and immunological reserves. This study hypothesizes that PNI, as a composite marker of albumin and lymphocytes, reflects both nutritional adequacy and immune competence, thereby predicting aGVHD outcomes independent of traditional risk factors. By elucidating this association, our findings aim to inform risk-adapted nutritional interventions and improve prognostic models in allo-HSCT care.

## Methods

### Patients and study design

**Figure 1:**
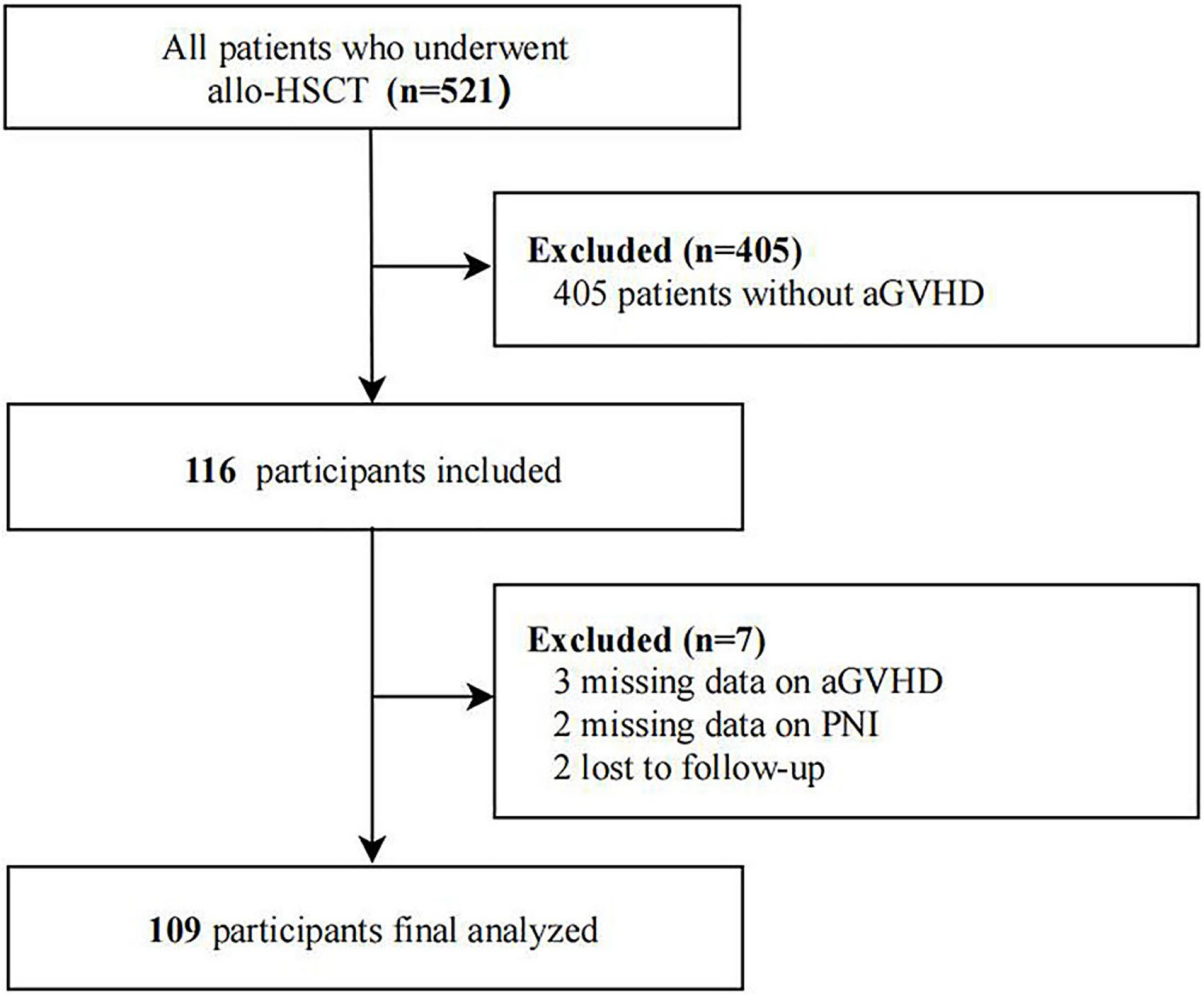
The flow chart of the study.

This retrospective study included 521 consecutive patients who underwent allo-HSCT at Henan Cancer Hospital from January 2019 to December 2024. Of these, 116 were diagnosed with aGVHD. Seven patients with incomplete data or who were lost to follow-up were excluded from the analysis. Ultimately, 109 patients with aGVHD were included in the cohort (Figure 1). This study followed a predefined analytical plan focused on PNI’s association with OS/EFS in aGVHD. All endpoints and covariates were specified prior to data extraction. The study was approved by the institutional review board of the Affiliated Cancer Hospital of Zhengzhou University & Henan Cancer Hospital (approval number: 2022–060-001). All procedures were carried out in accordance with the relevant guidelines and regulations, and the study was conducted in compliance with the Declaration of Helsinki.

### Conditioning regimens and GVHD prophylaxis

Recipients of unrelated donor and haploidentical transplants were treated with a regimen based on antihuman thymocyte immunoglobulin (ATG). In contrast, sibling allogeneic stem cell transplantation was performed without ATG. Conditioning regimens include myeloablative conditioning (MAC) and reduced-intensity conditioning (RIC) regimens. The MAC regimens consisted of Me-CCNU 250 mg/m^2^ on day −7, cytara-bine (Ara-C) 4 g/m^2^/day on days −6 to −5, busulfan (Bu) 3.2 mg/kg/day on days −6 to −4, and cyclophosphamide (Cy) 1.8 g/m^2^/day on days −3 to −2. The RIC regimens included fludarabine (120–180 mg/m^2^) with busulfan (≤8 mg/kg oral or 6.4 mg/kg i.v.) (Flu/Bu) or melphalan (≤150 mg/m2) (Flu/Mel). The choice of preparative regimen was based primarily on diagnosis, age, and tumor burden before HSCT. Patients with comorbidities and lower relapse risk might have been selected to receive RIC, whereas patients at higher relapse risk and with fewer comorbidities might have been selected to receive MAC. Our center’s protocol prioritizes aggressive GVHD prophylaxis reduction in high-risk malignancies to induce the graft-versus-leukemia (GVL) effect. The regimen included peripheral blood and bone marrow stem cell collection, ATG-based conditioning, and GVHD prophylaxis with methotrexate, mycophenolate mofetil (MMF), and cyclosporine A (CsA). CsA was administered intravenously at a dose of 2.5–3 mg/kg/day starting on day −5, continuing until bowel function normalized, after which oral CsA was initiated. Whole-blood CsA levels were monitored weekly by fluorescence polarization immunoassay (Cayman Chemical, Ann Arbor, MI), with the target concentration maintained between 150 and 250 ng/mL. If no GVHD was evident by day +180, CsA was tapered, except in SAA cases, where CsA was tapered after 1 year. MMF was given at 15 mg/kg every 12 h from day −5 to day +30, with the dose halved at day +30 and discontinued by day +60. Methotrexate was administered intravenously at 15 mg/m^2^ on day +1 and 10 mg/m^2^ on days +3, +6, and +11. In cases of aGVHD, patients received steroids (methylprednisolone 1 mg/kg/day). If the response to initial therapy was inadequate, patients were treated with anti-CD25 monoclonal antibody (basiliximab; Novartis AG, Basel, Switzerland).

### Definitions

aGVHD was assessed according to the modified Glucksberg criteria (20, 21). Overall survival (OS) was defined as the time from stem cell infusion to death from any cause. Event-free survival (EFS) was defined as the time from stem cell infusion to disease relapse/progression or death.

### PNI

The prognostic nutritional index (PNI) is a composite parameter combining serum albumin concentration and absolute lymphocyte count, reflecting the patient’s nutritional and immune status. It is used as an alternative marker to assess nutritional and inflammatory states as well as the host’s anti-tumor response, serving as a parameter that reflects the patient’s nutritional and immune conditions.

The PNI was calculated using serum albumin and absolute lymphocyte counts measured within 48 h of aGVHD diagnosis. Serum albumin was quantified using the Beckman Coulter AU5800 analyzer (Beckman Coulter, United States) via bromocresol green methodology. Absolute lymphocyte counts were derived from complete blood counts performed on a Sysmex XN-9000 hematology analyzer (Sysmex Corporation, Japan). The calculation formula is as follows: PNI = albumin (g/L) + 5 × lymphocyte count (×10^9/L) (22).

### Supportive treatment

All patients received prophylactic treatments to prevent infections. These included ganciclovir for viral infections, posaconazole or voriconazole for fungal infections, sulfamethoxazole for Pneumocystis carinii prevention, phenytoin for protection against central nervous system toxicity due to busulfan, mesylate to prevent hemorrhagic cystitis, and ursodeoxycholic acid to reduce the risk of hepatic vein occlusive disease. Additional supportive measures included hydration, urine alkalinization, irradiated blood component transfusions, and other care as needed. Subcutaneous recombinant human G-CSF and thrombopoietin were administered from day +4 until neutrophil and platelet engraftment. CMV and EBV viral loads were monitored biweekly during hospitalization. Anti-CMV therapy (ganciclovir and sodium phosphonate) was initiated when CMV DNA levels exceeded 1,000 copies/mL, while rituximab (100 mg) was given for EBV DNA levels ≥5,000 copies/mL on two consecutive tests.

### Statistical analysis

Histogram distribution, or Q–Q plot, or Kolmogorov–Smirnov test was used for determining whether variables were normally distributed. All normally distributed continuous variables were expressed presented as mean± SD, and skewed continuous variables were described as median (interquartile range [IQR]). Categorical variables were presented as frequencies (%). We used chi-square or Fisher’s exact (categorical variables), One-Way ANOVA test (normal distribution), or Kruskal–Wallis *H* test (skewed distribution) to test for differences among different PNI groups.

The effect of PNI on mortality was evaluated using Cox proportional hazards models (hazards ratio [HR] and 95% confidence interval [CI]) with adjustment for major covariables. The proportional hazard assumption was verified by inspection of “log–log” plots and by introducing interactions with survival time. Participants who were lost to follow-up were censored at that particular time point. Outcome of mortality was assessed with Kaplan–Meier survival curves according to the PNI (categorical) and evaluated with the log-rank test. PNI was entered as a categorical variable (three quantile). Patients were stratified into low (T1), intermediate (T2), and high (T3) PNI groups based on tertiles of the cohort distribution: T1, PNI (20.2–35.8); T2, PNI (35.8–42.5); T3, PNI (42.5–59.7). Tertile-based stratification was used, aligning with established methodologies in prognostic biomarker studies (11), to enable clinical risk stratification by distinguishing low-, intermediate-, and high-risk subgroups. This approach facilitates clinical risk stratification by distinguishing low-, intermediate-, and high-risk subgroups. We selected these confounders on the basis of judgment, previous scientific literature, all significant covariates in the univariate analysis, or their associations with the outcomes of interest or a change in effect estimate of more than 10%. We constructed 3 models: Model 1: Adjusted for Age and Sex; Model 2: Adjusted for Model 1 and Indication for HSCT, Stem cell sources, Type of transplantation, Conditioning regimen, Days from transplantation to diagnosis, ABO match, MNC count, CD34^+^ cells count; Model 3: Adjusted for Model 2 and Granulocyte implantation time, CMV viremia, EBV viremia, White blood cells, Hemoglobin and Platelets, Total bilirubin, Creatinine, Pulmonary infection, Intestinal infection, Febrile neutropenia, aGVHD grade. Tests for trend were conducted with multivariate regression models by entering the median value of each PNI tertile as a continuous variable in the models. Prespecified subgroup analyses were conducted according to Subgroup variables. Participants with missing data were excluded in the initial analysis, where all missing values were removed. We conducted a series of sensitivity analyses to evaluate the robustness of the findings of the study and how our conclusions can be affected by applying various association inference models. The calculated effect sizes and *p* values from all these models were reported and compared. Non-relapse mortality (NRM) was evaluated through competing risks analysis using the Aalen-Johansen estimator. Relapse-related mortality served as the competing event. Intergroup comparisons among PNI tertiles (T1/T2/T3) were performed with Gray’s subdistribution test. All analyses were performed using R Statistical Software (Version 4.2.2, http://www.R-project.org, The R Foundation) and Free Statistics analysis platform (Version 1.9, Beijing, China, http://www.clinicalscientists.cn/freestatistics). A two-sided *p*-value < 0.05 was considered statistically significant.

## Results

### Baseline characteristics

This study included 109 eligible patients with a mean age of 29.4 ± 15.3 years. During a follow-up period of 30 months, 69 deaths, 3 cases of disease relapse/progression, and 37 patients remained alive. Among the participants, 51 had Grade I-II aGVHD, and 58 had Grade III-IV aGVHD. Table 1 presents the general characteristics of the participants stratified by PNI levels. Significant differences were observed across the three groups in terms of white blood cells, hemoglobin, Platelets, time from stem cell infusion to death from any cause or relapse/progression or death and aGVHD grade (all *p* < 0.05).

**Table 1:** The baseline characteristics and demographic characteristics of the study population by categories of PNI.

Characteristic	PNI				
	Total (*n* = 109)	1 (*n* = 36)	2 (*n* = 36)	3 (*n* = 37)	*p*-value
Age, mean ± SD	29.4 ± 15.3	33.6 ± 16.5	29.8 ± 14.7	25.1 ± 13.7	0.059
Sex, *n* (%)					0.066
Male	68 (62.4)	28 (77.8)	20 (55.6)	20 (54.1)	
Female	41 (37.6)	8 (22.2)	16 (44.4)	17 (45.9)	
Indication for HSCT, *n* (%)					0.256
AML	29 (26.6)	8 (22.2)	7 (19.4)	14 (37.8)	
ALL	34 (31.2)	16 (44.4)	9 (25)	9 (24.3)	
MDS	16 (14.7)	3 (8.3)	7 (19.4)	6 (16.2)	
SAA	20 (18.3)	7 (19.4)	7 (19.4)	6 (16.2)	
Others	10 (9.2)	2 (5.6)	6 (16.7)	2 (5.4)	
Stem cell sources, *n* (%)					1
Bone marrow	103 (94.5)	34 (94.4)	35 (97.2)	34 (91.9)	
Peripheral blood	5 (4.6)	2 (5.6)	1 (2.8)	2 (5.4)	
Bone marrow + Peripheral blood	1 (0.9)	0 (0)	0 (0)	1 (2.7)	
Type of transplantation, *n* (%)					0.494
Unrelated match	50 (45.9)	20 (55.6)	16 (44.4)	14 (37.8)	
HLA match related	35 (32.1)	11 (30.6)	12 (33.3)	12 (32.4)	
Haplo-identical related	24 (22.0)	5 (13.9)	8 (22.2)	11 (29.7)	
Conditioning regimen, *n* (%)					0.359
MAC	56 (51.4)	17 (47.2)	22 (61.1)	17 (45.9)	
RIC	53 (48.6)	19 (52.8)	14 (38.9)	20 (54.1)	
ABO match, *n* (%)					0.67
Matched	42 (38.5)	16 (44.4)	13 (36.1)	13 (35.1)	
Unmatched	67 (61.5)	20 (55.6)	23 (63.9)	24 (64.9)	
Disease status before HSCT, *n* (%)^a^					0.723
CR	70 (64.2)	24 (66.7)	20 (55.6)	26 (70.3)	
PR or NR	18 (16.5)	5 (13.9)	8 (22.2)	5 (13.5)	
MRD before HSCT, *n* (%)^a,b^					0.966
Positive	68 (62.4)	23 (63.9)	21 (58.3)	24 (64.9)	
Negative	21 (19.3)	6 (16.7)	8 (22.2)	7 (18.9)	
Days from transplantation to diagnosis	49.0 (20.0, 100.0)	66.0 (19.8, 100.0)	27.5 (15.5, 80.8)	66.0 (39.0, 100.0)	0.079
MNC count, ×10^8^/Kg, mean ± SD	13.4 ± 6.5	13.2 ± 6.1	12.8 ± 6.5	14.1 ± 7.1	0.692
CD34^+^ cells count, ×10^6^/Kg, mean ± SD	6.8 ± 4.2	7.1 ± 3.6	7.0 ± 5.6	6.2 ± 3.1	0.616
Granulocyte implantation time, days, mean ± SD	12.5 ± 2.3	12.4 ± 2.2	12.3 ± 2.3	12.9 ± 2.3	0.486
CMV viremia, *n* (%)	24 (22.0)	9 (25)	5 (13.9)	10 (27)	0.348
EBV viremia, *n* (%)	18 (16.5)	6 (16.7)	7 (19.4)	5 (13.5)	0.792
Febrile neutropenia, *n* (%)	31 (28.4)	11 (30.6)	12 (33.3)	8 (21.6)	1.348
Pulmonary infection, *n* (%)	81 (74.3)	30 (83.3)	31 (86.1)	20 (54.1)	0.002
Intestinal infection, *n* (%)	58 (53.2)	23 (63.9)	21 (58.3)	14 (37.8)	0.063
White blood cells, ×10^9^/L, Median (IQR)	2.5 (1.1, 4.4)	2.3 (0.9, 3.5)	2.1 (1.0, 3.1)	3.6 (1.6, 5.6)	0.042
Hemoglobin, g/L, mean ± SD	87.2 ± 21.0	79.3 ± 21.5	85.1 ± 13.0	96.8 ± 23.3	< 0.001
Platelets, ×10^9^/L, median (IQR)	37.0 (22.0, 72.0)	27.5 (17.5, 45.0)	31.5 (21.8, 67.8)	65.0 (33.0, 91.0)	0.002
Total bilirubin, (μmol/L), median (IQR)	17.8 (11.2, 33.4)	31.4 (15.1, 62.4)	17.8 (11.3, 32.7)	12.9 (10.6, 21.0)	0.006
Creatinine (mmol/L), median (IQR)	51.0 (35.0, 84.0)	60.0 (35.0, 95.0)	50.0 (42.8, 83.0)	47.0 (31.0, 67.0)	0.422
Status, *n* (%)					0.226
Alive	37 (33.9)	11 (30.6)	15 (41.7)	11 (29.7)	
Death	69 (63.3)	25 (69.4)	21 (58.3)	23 (62.2)	
Disease relapse/progression	3 (2.8)	0 (0)	0 (0)	3 (8.1)	
NRM	66(60.6)	25 (69.4)	20 (55.6)	21(56.8)	
Time, months, median (IQR)					
From stem cell infusion to death from any cause	6.0 (3.0, 18.0)	5.0 (2.8, 7.2)	6.0 (3.8, 16.0)	12.0 (5.0, 26.0)	< 0.001
From stem cell infusion to relapse/progression or death	6.0 (3.0, 16.0)	5.0 (2.8, 7.2)	6.0 (3.8, 16.0)	12.0 (5.0, 23.0)	0.001
aGVHD, *n* (%)					0.003
Grade I–II	51 (46.8)	9 (25)	18 (50)	24 (64.9)	
Grade III–IV	58 (53.2)	27 (75)	18 (50)	13 (35.1)	

^a^Indicates that patients with *n* = 20 SAA were excluded from the MRD and disease status statistics before HSCT.

^b^MRD was measured by flow cytometry before HSCT and MRD levels≥10^−4^ was defined as positive.

PNI, prognostic nutritional index; T1, PNI (20.2–35.8); T2, PNI (35.8–42.5); T3, PNI(42.5–59.7); SD, standard deviation; IQR, interquartile range; AML, acute myelocytic leukemia; ALL, acute lymphocytic leukemia; MDS, myelodysplastic syndrome; SAA, severe aplastic anemia; MAC, myeloablative conditioning; RIC, reduced-intensity conditioning; CR, complete remission; PR, partial remission; NR, non-remission; MNC, mononuclear cell count; MRD: minimal residual disease; CMV, cytomegalovirus; EBV, EB virus; NRM, non-relapse mortality; aGVHD, acute graft-versus-host disease.

### Associations between PNI and prognosis

**Figure 2:**
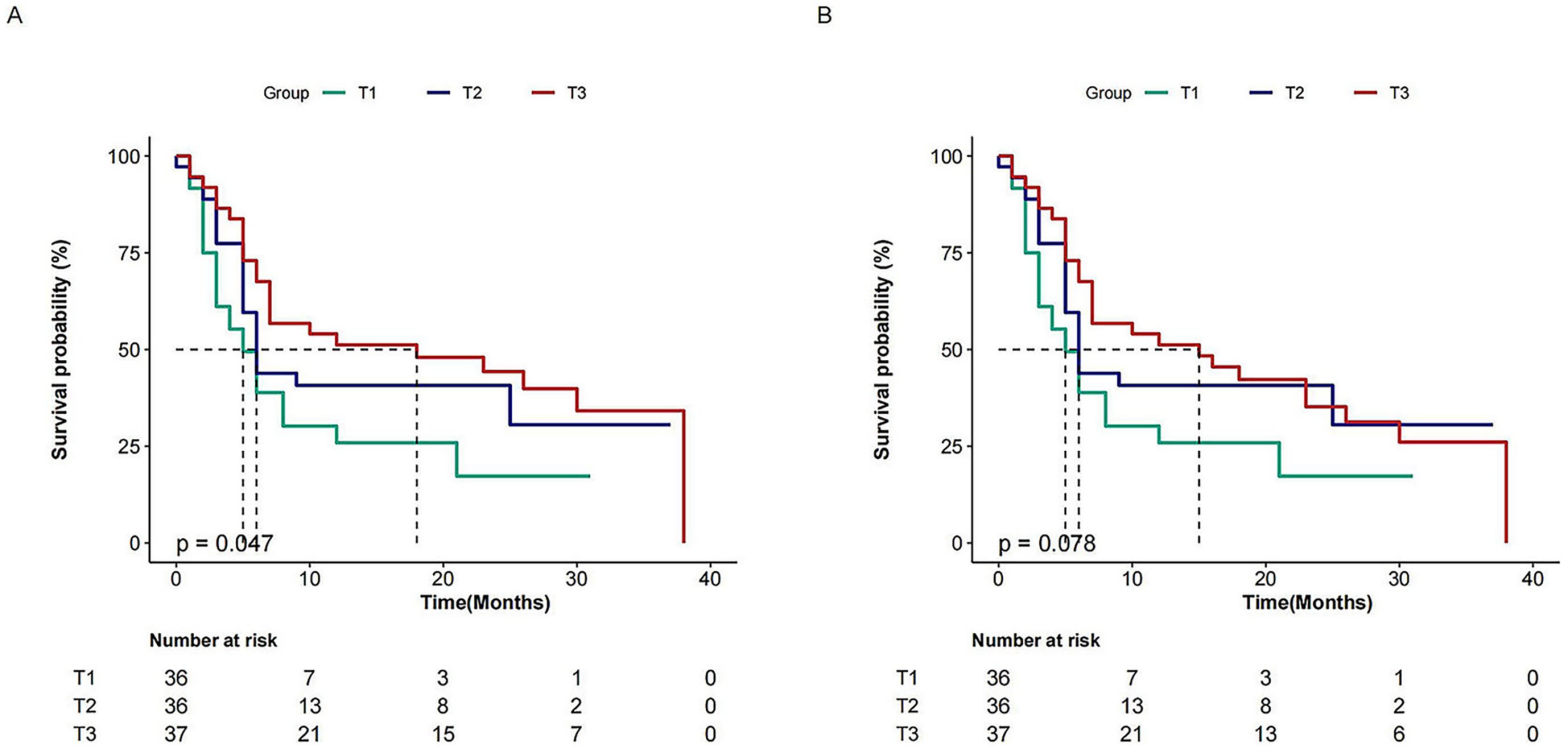
Kaplan–Meier survival curves depicting survival rates and the percentage (%) of patients with aGVHD after allo-HSCT, categorized by different PNI levels.

Table 2 presents the results of a multivariable Cox regression analysis exploring the association between PNI and OS as well as EFS. A high PNI as a continuous variable was associated with an increased OS (HR, 0.95; 95% CI, 0.88–0.98; *p* = 0.018) and EFS (HR, 0.96; 95% CI, 0.88 ~ 0.97; *p* = 0.035) after adjusting for age, sex, stem cell sources, type of transplantation, conditioning regimen, ABO match, MNC count, CD34^+^ cells count, granulocyte implantation time, CMV viremia, EBV viremia, white blood cells, hemoglobin and platelets, total bilirubin, creatinine, pulmonary infection, intestinal infection, febrile neutropenia, aGVHD grade. When PNI was evaluated as a categorical variable, the adjusted hazard ratios (HR) for PNI and OS in T2 (35.8–42.5) and T3 (42.5–59.7) were 0.52 (95% CI: 0.23–0.86, *p* = 0.049) and 0.43 (95% CI: 0.18–0.73, *p* = 0.013), respectively, compared to individuals with lower T1 (20.2–35.8). For EFS, the adjusted HR values for PNI in T2 (35.8–42.5) and T3 (42.5–59.7) were 0.32 (95% CI: 0.17–0.59, *p* = 0.026) and 0.31 (95% CI: 0.16–0.56, *p* = 0.018), respectively, when compared to those with lower T1 (20.2–35.8). These results suggest a potential association between lower PNI levels and poorer prognosis. Kaplan–Meier analysis demonstrated poorer OS (*p* = 0.047) in the lower PNI group. Although not reaching conventional statistical significance (*p* = 0.078), the EFS trend toward poorer outcomes in Group T3, combined with consistent hazard ratios and mechanistic plausibility, merits further investigation (Figure 2).

**Table 2:** Association between PNI and mortality in patients with aGVHD following allo-HSCT.

Characteristic	Total, *n* (%)	Event, *n* (%)	Crude model		Model 1		Model 2		Model 3	
			HR (95%CI)	*p*-value	HR (95%CI)	*p*-Value	HR (95%CI)	*p*-Value	HR (95%CI)	*p*-Value
OS										
PNI	109	69 (63.3)	0.96 (0.92 ~ 0.99)	0.008	0.96 (0.92 ~ 0.99)	0.01	0.94 (0.9 ~ 0.97)	<0.001	0.95 (0.88 ~ 0.98)	0.018
PNI category										
T1	36	25 (69.4)	1(Ref)		1(Ref)		1(Ref)		1(Ref)	
T2	36	21 (58.3)	0.65 (0.36 ~ 1.16)	0.141	0.65 (0.36 ~ 1.17)	0.147	0.4 (0.2 ~ 0.81)	0.011	0.52 (0.23 ~ 0.86)	0.049
T3	37	23 (62.2)	0.49 (0.27 ~ 0.88)	0.017	0.49 (0.27 ~ 0.9)	0.022	0.28 (0.13 ~ 0.59)	0.001	0.43 (0.18 ~ 0.73)	0.013
Trend test				0.018		0.023		0.001		0.023
EFS										
PNI	109	72 (66.1)	0.96 (0.93 ~ 0.99)	0.017	0.96 (0.93 ~ 0.99)	0.019	0.94 (0.91 ~ 0.97)	0.001	0.96 (0.88 ~ 0.97)	0.035
PNI category										
T1	36	25 (69.4)	1(Ref)		1(Ref)		1(Ref)		1(Ref)	
T2	36	21 (58.3)	0.63 (0.35 ~ 1.12)	0.116	0.63 (0.35 ~ 1.13)	0.118	0.36 (0.18 ~ 0.73)	0.005	0.32 (0.17 ~ 0.59)	0.026
T3	37	26 (70.3)	0.54 (0.31 ~ 0.95)	0.033	0.54 (0.3 ~ 0.97)	0.04	0.31 (0.15 ~ 0.64)	0.002	0.31 (0.16 ~ 0.56)	0.018
Trend test				0.038		0.044		0.003		0.017

PNI, prognostic nutritional index; T1, PNI (20.15–35.8); T2, PNI (35.8–42.5); T3, PNI (42.5–59.7); aGVHD, acute graft-versus-host disease; allo-HSCT, allogeneic hematopoietic stem cell transplantation; HR, Hazard Ratio; CI, confidence interval; Ref, reference; OS, overall survival; EFS, event-free survival.

Model 1: Adjusted for age and sex.

Model 2: Adjusted for model 1 and Indication for HSCT, stem cell sources, type of transplantation, conditioning regimen, days from transplantation to diagnosis, ABO match, MNC count, CD34^+^ cells count.

Model 3: Adjusted for Model 2 and granulocyte implantation time, CMV viremia, EBV viremia, white blood cells, hemoglobin and platelets, Total bilirubin, creatinine, pulmonary infection, intestinal infection, febrile neutropenia, aGVHD grade.

The cumulative mortality profiles stratified by PNI tertiles (T1-T3) demonstrated significant divergence (Gray’s test, *p* = 0.048). The T1 cohort (lowest PNI) exhibited the highest NRM burden, reaching 70% at 10 months (95% CI: 8.2–14.1). Strikingly, T3 (highest PNI) maintained the most favorable profile with only 50% NRM at 25 months (95% CI: 20.1–36.5). The T2 cohort displayed intermediate outcomes: 50.5% (95% CI: 16.7–31.2), 52.2% (95% CI: 37.1–55.8), and 62.2% (95% CI: 52.3–72.9) at 10/20/30 months, respectively. Risk set analysis revealed drastic attrition in T1 (*n* = 36 to 1 at 25 months) versus sustained retention in T3 (*n* = 37 to 22 at 30 months) (Supplementary Figure 1).

Moreover, the 1-year cumulative incidence of non-relapse mortality (NRM) was 41.7% (95% CI: 0.33–0.53). In multivariable analysis, the group with lower PNI levels (T1: 20.2–35.8) was associated with a significantly higher risk of NRM compared to the groups with higher PNI levels (T2/T3). Specifically, relative to the T1 group, the HR for NRM in the T2 (35.8–42.5) group was 0.41 (95% CI: 0.19–0.88; *p* = 0.023) and in the T3 (42.5–59.7) group was 0.34 (95% CI: 0.14–0.82; *p* = 0.017), demonstrating significantly lower NRM risk in both higher PNI groups.

### Subgroup and interaction analyses

**Figure 3:**
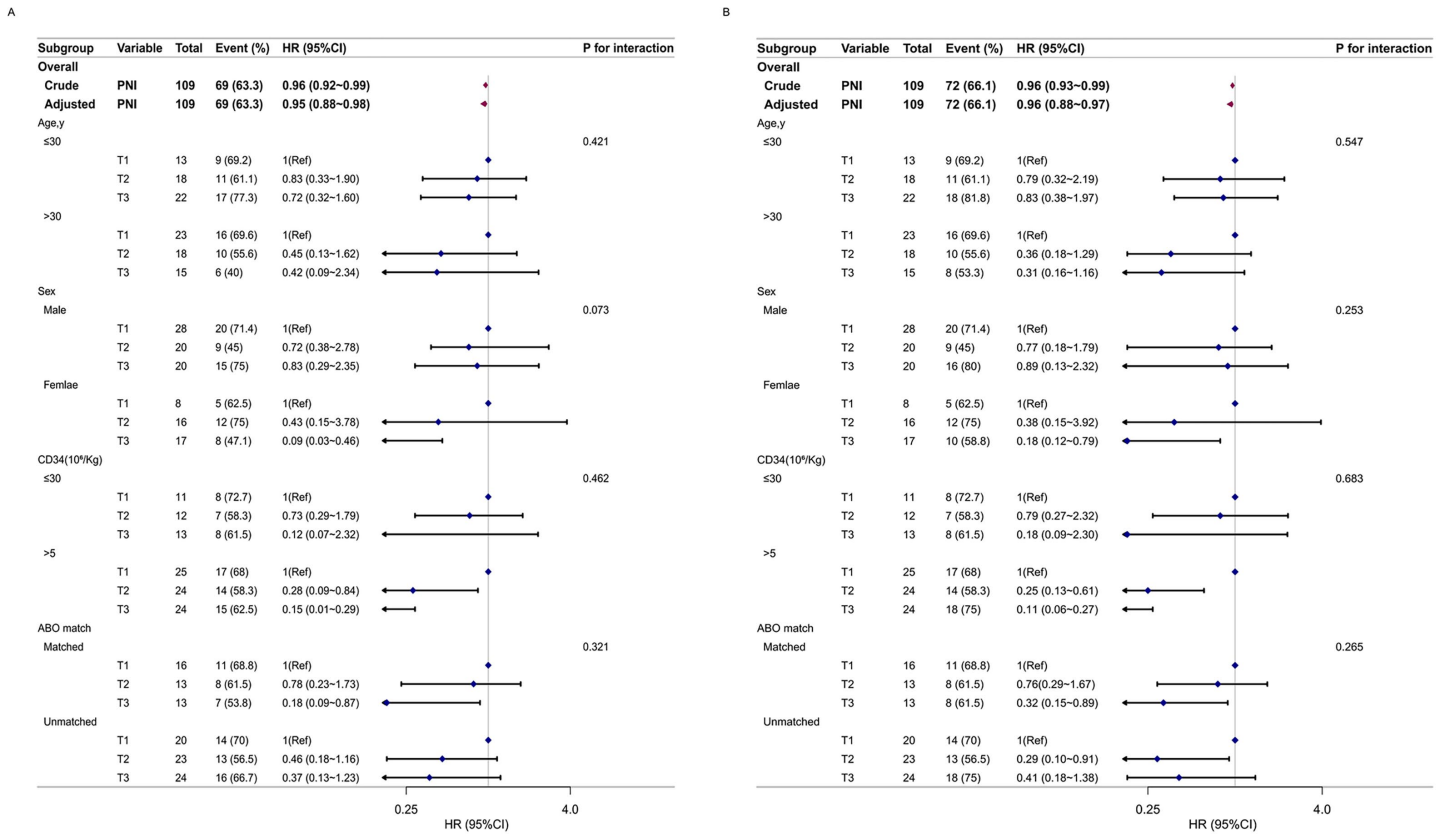
The relationship between PNI and prognosis based on basic characteristics. For each stratification factor, all other variables (age, sex, indication for HSCT, stem cell source, type of transplantation, conditioning regimen, ABO match, days from transplantation to diagnosis, MNC count, CD34+ cell count, granulocyte infusion time, CMV viremia, EBV viremia, white blood cell count, hemoglobin, and platelet count, total bilirubin, creatinine, pulmonary infection, intestinal infection, febrile neutropenia, aGVHD grade) were adjusted.

Subgroup and interaction analyses were conducted to assess the stability of the association between PNI levels and OS and EFS (Figure 3), after adjusting for all covariates included in Model 3. Subgroup analyses based on age, sex, ABO match and CD34^+^ cells count revealed no statistically significant interactions (all interaction *p*-values > 0.05). Additionally, we performed subgroup and interaction analyses concerning transplant modalities, conditioning regimens, and infection, all of which yielded robust results. Due to space constraints in the main manuscript, these subgroup analyses are presented in Supplementary Tables 2, 3.

The results from both the subgroup and interaction analyses confirmed the stability of the association between PNI levels and OS and EFS.

### Sensitivity analyses

After excluding patients with a survival time of less than 3 months, subsequent sensitivity analyses confirmed that the association between PNI levels and OS and EFS remained consistent (Supplementary Table 1). A high PNI as a continuous variable was associated with an increased OS (HR, 0.91; 95% CI, 0.86–0.97; *p* = 0.014) and EFS [HR, 0.91; 95% CI, (0.81 ~ 0.96); *p* = 0.038] after adjusting for age, sex, stem cell sources, type of transplantation, conditioning regimen, ABO match, MNC count, CD34^+^ cells count, granulocyte implantation time, CMV viremia, EBV viremia, white blood cells, hemoglobin and platelets, total bilirubin, creatinine, pulmonary infection, intestinal infection, febrile neutropenia, aGVHD grade. When PNI was evaluated as a categorical variable, the adjusted HR for PNI and OS in T2 (35.8–42.5) and T3 (42.5–59.7) were 0.43 (95% CI: 0.12–0.69, *p* = 0.045) and 0.37 (95% CI: 0.12–0.96, *p* = 0.032), respectively, compared to individuals with lower T1 (20.2–35.8) PNI levels. For EFS, the adjusted HRs for PNI in T2 (35.8–42.5) and T3 (42.5–59.7) were 0.33 (95% CI: 0.09–0.79, *p* = 0.021) and 0.29 (95% CI: 0.08–0.67, *p* = 0.014), respectively.

## Discussion

This retrospective cohort study examined the association between PNI levels and mortality in patients with aGVHD following allo-HSCT. The findings revealed that lower PNI levels were significantly correlated with a higher risk of mortality, highlighting its potential as a valuable prognostic biomarker for these patients. No significant interaction was observed between PNI and mortality, further validating the reliability and robustness of our results. PNI could serve as an easily measurable marker to predict outcomes, enabling early identification of high-risk patients and timely interventions to improve prognosis.

The GVHD is a serious complication following HSCT, where donor immune cells attack the host tissues, closely related to immune regulation imbalance (23). Current studies show that prevention strategies for GVHD (such as cyclophosphamide) need to balance immunosuppression and antitumor effects (9). Additionally, a patient’s nutritional status may indirectly modulate GVHD risk by affecting immune function (24). Inflammation and malnutrition have been confirmed to be associated with poor GVHD prognosis. Lymphocyte count (a component of PNI, reflecting inflammatory status) is associated with exposure to anti-thymocyte globulin (ATLG) and may influence the occurrence of GVHD (25). Low albumin levels (a key component of PNI), reflecting malnutritional status, are associated with the incidence and severity of aGVHD (26, 27). PNI, as a comprehensive indicator of nutritional status and immune function, holds potential prognostic value in HSCT patients (28). Studies show that pre-transplant nutritional status (including PNI) significantly impacts post-transplant survival outcomes (29). In HSCT, low PNI or low albumin levels are associated with poorer progression-free survival (PFS) and overall survival (OS) (26, 30, 31). A retrospective analysis of allo-HSCT patients found that PNI is an independent predictor of non-relapse mortality (NRM) (32).

This study investigates the prognostic value of PNI levels in patients with acute graft-versus-host disease (aGVHD) following allo-HSCT, a relatively underexplored area. As a cost-effective and widely accessible biomarker, PNI is well-suited for clinical application and has the potential to enhance existing prognostic models, supporting the development of future composite biomarker strategies. The focus of this study is specifically on the aGVHD population, offering valuable insights into the clinical utility of PNI in predicting mortality risk. While prior research has established associations between PNI and various cancers as well as systemic inflammatory diseases, our study is unique in its concentration on aGVHD, a complex complication post-HSCT. By highlighting the prognostic significance of PNI in this cohort, we provide a novel perspective on its potential to inform early interventions and optimize treatment strategies in the context of HSCT outcomes.

Current prognostic models for aGVHD primarily rely on clinical grading systems (such as Glucksberg and IBMTR) or inflammatory markers (e.g., ST2, REG3α) (33, 34), but there is a lack of accessible, low-cost serum biomarkers. PNI, a routine and cost-effective biochemical marker, is the first to be systematically assessed for its independent prognostic significance in aGVHD patients, highlighting its potential for clinical application. Monitoring PNI levels may serve as an indicator of aGVHD severity, providing a biological foundation for prognosis evaluation. In our retrospective cohort study, we adjusted for confounding variables (such as age, donor type, and GVHD prevention regimen) using multivariate Cox regression models, confirming PNI’s independent association with mortality risk. Subgroup analyses were conducted to ensure the robustness of our findings and minimize bias. This study represents the first demonstration of PNI’s role in prognostic stratification for aGVHD, establishing a basis for the development of future biomarker-based models. Moreover, the strong association between PNI and NRM but not relapse mortality underscores PNI’s value in predicting vulnerability to transplant-related complications rather than disease recurrence.

As a retrospective study, this research is susceptible to selection bias and confounding factors. Differences in baseline patient characteristics, transplant types, conditioning regimens, and other variables may impact the results. Furthermore, the timing and frequency of PNI measurements might affect the precision and dependability of the findings. Although we adjusted for key transplant-related variables, data on infection severity scores and detailed GVHD treatment regimens (such as corticosteroid dosage and second-line agents) were not uniformly available. Future studies will incorporate these confounding factors to refine prognostic models. The retrospective design inherently carries risks of unmeasured confounding. Prospective validation with registered protocols (e.g., ClinicalTrials.gov) is warranted. The elevated grade III-IV aGVHD incidence (53.2%) reflects our center’s high-risk profile (MAC/HLA-mismatched recipients) and aggressive GvHD prophylaxis reduction, potentially limiting generalizability to centers with different strategies. The predominance of younger patients (mean age 29.4 ± 15.3 years; 38.5% pediatric) may limit the generalizability of our findings to older allo-HSCT populations. To address these limitations, future prospective studies are necessary to more effectively confirm the relationship between PNI levels and mortality outcomes in aGVHD patients, while accounting for these confounding variables.

Based on the study’s results, future investigations could focus on evaluating the combined use of PNI with other relevant biomarkers, including inflammatory markers and cytokines, to refine the prognostic accuracy for aGVHD. Moreover, examining the temporal fluctuations in PNI levels could offer critical insights into their long-term effects on patient outcomes. In addition, exploring the underlying molecular pathways by which PNI affects the initiation and progression of aGVHD may lead to the identification of novel therapeutic targets, ultimately advancing treatment strategies.

## Conclusion

In this cohort of patients with aGVHD following allo-HSCT, our study underscores the association between lower PNI levels and poorer prognosis. The findings presented in this study warrant attention and should be considered in the development and updating of clinical guidelines. Given the potential for confounding, further research is necessary to validate these results.

## Data Availability

The raw data supporting the conclusions of this article will be made available by the authors, without undue reservation.

## Supplementary material

SUPPLEMENTARY FIGURE 1 Cumulative incidence of mortality stratified by PNI tertiles.
