# Supplementary figures and images for: Association between prognostic nutritional index and prognosis in acute graft-versus-host disease following allogeneic hematopoietic stem cell transplantation: a retrospective cohort study
Source: Front Nutr. 2025 Nov 28;12:1661993. doi: 10.3389/fnut.2025.1661993 (PMC12698373; doi:10.3389/fnut.2025.1661993)

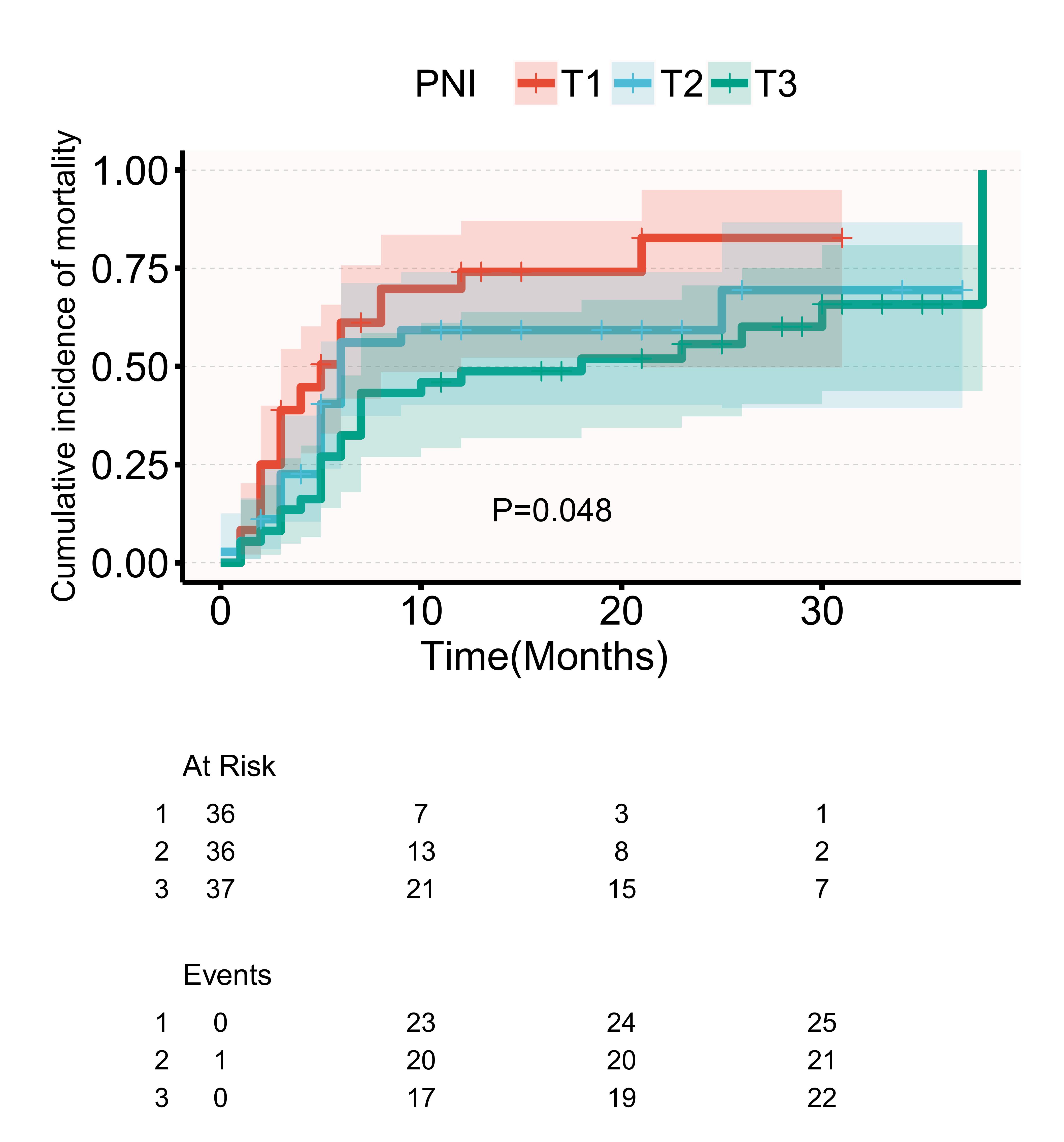

Supplement: SUPPLEMENTARY FIGURE 1 — Cumulative incidence of mortality stratified by PNI tertiles. [file Image_1.jpeg]
