# Supplementary material for: Association between prognostic nutritional index and prognosis in acute graft-versus-host disease following allogeneic hematopoietic stem cell transplantation: a retrospective cohort study
Source: Front Nutr. 2025 Nov 28;12:1661993. doi: 10.3389/fnut.2025.1661993 (PMC12698373; doi:10.3389/fnut.2025.1661993)
Supplement: Supplementary file 2 [file Table_1.docx]

**Supplementary Table 1.** Association betweenPNI and prognosis in patients with aGVHD

following allo-HSCT, excluding those with a survival time of less than 3 months

| Characteristic | Total,  n (%) | Event,  n (%) | Crude model | |  | Model 1 | |  | Model 2 | |  | Model 3 | |
| --- | --- | --- | --- | --- | --- | --- | --- | --- | --- | --- | --- | --- | --- |
|  |  |  | HR (95%CI) | *p*-Value |  | HR (95%CI) | *p*-Value |  | HR (95%CI) | *p*-Value |  | HR (95%CI) | *p*-Value |
| OS |  |  |  |  |  |  |  |  |  |  |  |  |  |
| PNI | 80 | 38 (47.5) | 0.96 (0.92~1) | 0.082 |  | 0.93 (0.89~0.98) | 0.004 |  | 0.92 (0.88~0.97) | 0.003 |  | 0.91 (0.86~0.97) | 0.014 |
| PNI category |  |  |  |  |  |  |  |  |  |  |  |  |  |
| T1 | 21 | 10 (47.6) | 1(Ref) |  |  | 1(Ref) |  |  | 1(Ref) |  |  | 1(Ref) |  |
| T2 | 27 | 14 (51.9) | 0.52 (0.22~1.21) | 0.129 |  | 0.45 (0.18~1.1) | 0.081 |  | 0.58 (0.20~1.68) | 0.313 |  | 0.43  (0.12~0.69) | 0.045 |
| T3 | 32 | 14 (43.8) | 0.26 (0.11~0.61) | 0.002 |  | 0.22 (0.09~0.56) | 0.001 |  | 0.31 (0.10~0.92) | 0.034 |  | 0.37 (0.12~0.96) | 0.032 |
| Trend test |  | 38 (47.5) |  | 0.002 |  |  | 0.307 |  |  | 0.03 |  |  | 0.043 |
| EFS |  |  |  |  |  |  |  |  |  |  |  |  |  |
| PNI | 80 | 45 (56.2) | 0.97 (0.93~1.01) | 0.152 |  | 0.97 (0.93~1.01) | 0.12 |  | 0.93 (0.88~0.98) | 0.007 |  | 0.91 (0.81~0.96) | 0.038 |
| PNI category |  |  |  |  |  |  |  |  |  |  |  |  |  |
| T1 | 21 | 11 (52.4) | 1(Ref) |  |  | 1(Ref) |  |  | 1(Ref) |  |  | 1(Ref) |  |
| T2 | 27 | 13 (48.1) | 0.77 (0.34~1.72) | 0.522 |  | 0.78 (0.35~1.74) | 0.537 |  | 0.46 (0.16~1.32) | 0.148 |  | 0.33  (0.09~0.79) | 0.021 |
| T3 | 32 | 21 (65.6) | 0.76 (0.36~1.61) | 0.471 |  | 0.71 (0.33~1.54) | 0.386 |  | 0.33 (0.11~0.98) | 0.045 |  | 0.29 (0.08~0.67) | 0.014 |
| Trend test |  |  |  | 0.515 |  | 0.85 (0.58~1.25) | 0.406 |  |  | 0.058 |  |  | 0.015 |

PNI, Prognostic Nutritional Index; T1, PNI (20.15-35.8); T2, PNI (35.8-42.5); T3, PNI(42.5-59.7); aGVHD, acute graft-versus-host disease; allo-HSCT, allogeneic hematopoietic stem cell transplantation; HR, Hazard Ratio; CI, Confidence Interval; Ref, reference; OS, overall survival; EFS, event-free survival.

Model 1: Adjusted for Age and Sex;

Model2: Adjusted for Model1 and Indication for HSCT, Stem cell sources, Type of transplantation, Conditioning regimen, Days from transplantation to diagnosis, ABO match, MNC count, CD34^+^ cells count;

Model3: Adjusted for Model2 and Granulocyte implantation time, CMV viremia, EBV viremia, White blood cells, Hemoglobin and Platelets, Total bilirubin, Creatinine, Pulmonary infection, Intestinal infection, Febrile neutropenia, aGVHD grade.

.

.
