# Supplementary material for: Association between prognostic nutritional index and prognosis in acute graft-versus-host disease following allogeneic hematopoietic stem cell transplantation: a retrospective cohort study
Source: Front Nutr. 2025 Nov 28;12:1661993. doi: 10.3389/fnut.2025.1661993 (PMC12698373; doi:10.3389/fnut.2025.1661993)
Supplement: Supplementary file 3 [file Table_2.docx]

**Supplementary Table 2**  Subgroup analyses of the association between PNI and OS in aGVHD following allo-HSCT

| Subgroup | Variable | OS | | | *P*-Value | *P* for interaction |
| --- | --- | --- | --- | --- | --- | --- |
|  |  | Total | Event(%) | HR(95%CI) |  |  |
| Type of transplantation |  |  |  |  |  |  |
| Unrelated match |  |  |  |  |  | 0.177 |
|  | T1 | 20 | 16 (80) | 1(Ref) |  |  |
|  | T2 | 16 | 8 (50) | 0.24 (0.07~0.91) | 0.035 |  |
|  | T3 | 14 | 10 (71.4) | 0.27 (0.09~0.82) | 0.021 |  |
| HLA match related |  |  |  |  |  |  |
|  | T1 | 11 | 5 (45.5) | 1(Ref) |  |  |
|  | T2 | 12 | 6 (50) | 0.53 (0.04~7.46) | 0.637 |  |
|  | T3 | 12 | 8 (66.7) | 0.26 (0.03~2.09) | 0.204 |  |
| Haplo-identical related |  |  |  |  |  |  |
|  | T1 | 5 | 4 (80) | 1(Ref) |  |  |
|  | T2 | 8 | 7 (87.5) | 0.81 (0.29~3.51) | <0.001 |  |
|  | T3 | 11 | 5 (45.5) | 0.27 (0.07~1.12) | <0.001 |  |
| Conditioning regimen |  |  |  |  |  |  |
| MAC |  |  |  |  |  | 0.149 |
|  | T1 | 17 | 9 (52.9) | 1(Ref) |  |  |
|  | T2 | 22 | 13 (59.1) | 0.88 (0.33~3.57) | 0.894 |  |
|  | T3 | 17 | 13 (76.5) | 0.61 (0.19~1.98) | 0.414 |  |
| RIC |  |  |  |  |  |  |
|  | T1 | 19 | 16 (84.2) | 1(Ref) |  |  |
|  | T2 | 14 | 8 (57.1) | 0.35 (0.07~1.81) | 0.208 |  |
|  | T3 | 20 | 10 (50) | 0.09 (0.02~0.43) | 0.002 |  |
| CMV viremia |  |  |  |  |  |  |
| No |  |  |  |  |  | 0.126 |
|  | T1 | 27 | 16 (59.3) | 1(Ref) |  |  |
|  | T2 | 31 | 18 (58.1) | 0.81 (0.36~1.83) | 0.61 |  |
|  | T3 | 27 | 16 (59.3) | 0.58 (0.23~1.45) | 0.248 |  |
| Yes |  |  |  |  |  |  |
|  | T1 | 9 | 9 (100) | 1(Ref) |  |  |
|  | T2 | 5 | 3 (60) | 0.38 (0.10~1.42) | <0.001 |  |
|  | T3 | 10 | 7 (70) | 0.21 (0.07~0.62) | <0.001 |  |
| EBV viremia |  |  |  |  |  |  |
| No |  |  |  |  |  | 0.347 |
|  | T1 | 30 | 20 (66.7) | 1(Ref) |  |  |
|  | T2 | 29 | 18 (62.1) | 0.62 (0.27~1.34) | 0.211 |  |
|  | T3 | 32 | 19 (59.4) | 0.24 (0.11~0.56) | 0.001 |  |
| Yes |  |  |  |  |  |  |
|  | T1 | 6 | 5 (83.3) | 1(Ref) |  |  |
|  | T2 | 7 | 3 (42.9) | 0.35 (0.17~1.43) | <0.001 |  |
|  | T3 | 5 | 4 (80) | 0.69 (0.26~2.34) | <0.001 |  |
| Febrile neutropenia |  |  |  |  |  |  |
| No |  |  |  |  |  | 0.12 |
|  | T1 | 24 | 17 (70.8) | 1(Ref) |  |  |
|  | T2 | 23 | 14 (60.9) | 0.67 (0.31~1.45) | 0.54 |  |
|  | T3 | 25 | 14 (56) | 0.18 (0.06~0.5) | 0.001 |  |
| Yes |  |  |  |  |  |  |
|  | T1 | 12 | 8 (66.7) | 1(Ref) |  |  |
|  | T2 | 13 | 7 (53.8) | 0.24 (0.03~1.9) | 0.175 |  |
|  | T3 | 12 | 9 (75) | 0.86 (0.08~9.4) | 0.899 |  |

PNI, Prognostic Nutritional Index; OS, overall survival; aGVHD, acute graft-versus-host disease; allo-HSCT, allogeneic hematopoietic stem cell transplantation; MAC, myeloablative conditioning; RIC, reduced-intensity conditioning.
