# Supplementary material for: Association between prognostic nutritional index and prognosis in acute graft-versus-host disease following allogeneic hematopoietic stem cell transplantation: a retrospective cohort study
Source: Front Nutr. 2025 Nov 28;12:1661993. doi: 10.3389/fnut.2025.1661993 (PMC12698373; doi:10.3389/fnut.2025.1661993)
Supplement: Supplementary file 4 [file Table_3.docx]

**Supplementary Table 3**  Subgroup analyses of the association between PNI and EFS in aGVHD following allo-HSCT

| Subgroup | Variable | EFS | | | *P*-Value | *P* for interaction |
| --- | --- | --- | --- | --- | --- | --- |
|  |  | Total | Event(%) | HR(95%CI) |  |  |
| Type of transplantation |  |  |  |  |  | 0.062 |
| Unrelated match | T1 | 20 | 16 (80) | 1(Ref) |  |  |
|  | T2 | 16 | 8 (50) | 0.24 (0.06~0.88) | 0.032 |  |
|  | T3 | 14 | 11 (78.6) | 0.33 (0.09~1.27) | 0.108 |  |
| HLA match related |  |  |  |  |  |  |
|  | T1 | 11 | 5 (45.5) | 1(Ref) |  |  |
|  | T2 | 12 | 6 (50) | 0.01 (0~0.38) | 0.014 |  |
|  | T3 | 12 | 10 (83.3) | 0.54 (0.02~13.31) | 0.705 |  |
| Haplo-identical related |  |  |  |  |  |  |
|  | T1 | 5 | 4 (80) | 1(Ref) |  |  |
|  | T2 | 8 | 7 (87.5) | 0.88 (0.39~4.57) | <0.001 |  |
|  | T3 | 11 | 5 (45.5) | 0.34 (0.17~2.15) | <0.001 |  |
| Conditioning regimen |  |  |  |  |  | 0.059 |
| MAC |  |  |  |  |  |  |
|  | T1 | 17 | 9 (52.9) | 1(Ref) |  |  |
|  | T2 | 22 | 13 (59.1) | 0.79 (0.23~2.77) | 0.513 |  |
|  | T3 | 17 | 14 (82.4) | 0.63 (0.31~2.56) | 0.601 |  |
| RIC |  |  |  |  |  |  |
|  | T1 | 19 | 16 (84.2) | 1(Ref) |  |  |
|  | T2 | 14 | 8 (57.1) | 0.07 (0.01~0.42) | 0.004 |  |
|  | T3 | 20 | 12 (60) | 0.07 (0.01~0.36) | 0.001 |  |
| CMV viremia |  |  |  |  |  | 0.260 |
| No |  |  |  |  |  |  |
|  | T1 | 27 | 16 (59.3) | 1(Ref) |  |  |
|  | T2 | 31 | 18 (58.1) | 0.60 (0.25~1.45) | 0.261 |  |
|  | T3 | 27 | 18 (66.7) | 0.61 (0.23~1.61) | 0.319 |  |
| Yes |  |  |  |  |  |  |
|  | T1 | 9 | 9 (100) | 1(Ref) |  |  |
|  | T2 | 5 | 3 (60) | 0.43 (0.23~2.56) | <0.001 |  |
|  | T3 | 10 | 8 (80) | 0.39 (0.09~1.39) | <0.001 |  |
| EBV viremia |  |  |  |  |  | 0.605 |
| No |  |  |  |  |  |  |
|  | T1 | 30 | 20 (66.7) | 1(Ref) |  |  |
|  | T2 | 29 | 18 (62.1) | 0.39 (0.17~0.91) | 0.03 |  |
|  | T3 | 32 | 22 (68.8) | 0.24 (0.09~0.66) | 0.005 |  |
| Yes |  |  |  |  |  |  |
|  | T1 | 6 | 5 (83.3) | 1(Ref) |  |  |
|  | T2 | 7 | 3 (42.9) | 0.39 (0.09~2.89) | <0.001 |  |
|  | T3 | 5 | 4 (80) | 0.67 (0.19~1.98) | <0.001 |  |
| Febrile neutropenia |  |  |  |  |  | 0.453 |
| No |  |  |  |  |  |  |
|  | T1 | 25 | 19 (76) | 1(Ref) |  |  |
|  | T2 | 24 | 14 (58.3) | 0.2 (0.07~0.57) | 0.002 |  |
|  | T3 | 29 | 19 (65.5) | 0.29 (0.1~0.8) | 0.017 |  |
| Yes |  |  |  |  |  |  |
|  | T1 | 11 | 6 (54.5) | 1(Ref) |  |  |
|  | T2 | 12 | 7 (58.3) | 0.56 (0.34~2.89) | 0.006 |  |
|  | T3 | 8 | 7 (87.5) | 0.39(0.18~1.79) | 0.064 |  |

PNI, Prognostic Nutritional Index; EFS, event-free survival; aGVHD, acute graft-versus-host disease; allo-HSCT, allogeneic hematopoietic stem cell transplantation; MAC, myeloablative conditioning; RIC, reduced-intensity conditioning.
