# Supplementary material for: Association between prognostic nutritional index and prognosis in acute graft-versus-host disease following allogeneic hematopoietic stem cell transplantation: a retrospective cohort study
Source: Front Nutr. 2025 Nov 28;12:1661993. doi: 10.3389/fnut.2025.1661993 (PMC12698373; doi:10.3389/fnut.2025.1661993)
Supplement: Supplementary file 5 [file Table_4.docx]

Supplemental Table 4. Immunosuppressant use in aGVHD patients (n=109)

| aGVHD Grade | Steroids Only | Steroids + Recombinant Humanized Anti-CD25 Monoclonal Antibody Injection |
| --- | --- | --- |
| I-II (n=51) | 48 (94.1%) | 3 (5.9%) |
| III-IV (n=58) | 34 (58.6%) | 24 (41.4%) |

aGVHD, acute graft-versus-host disease.
