# Supplementary material for: Association between prognostic nutritional index and prognosis in acute graft-versus-host disease following allogeneic hematopoietic stem cell transplantation: a retrospective cohort study
Source: Front Nutr. 2025 Nov 28;12:1661993. doi: 10.3389/fnut.2025.1661993 (PMC12698373; doi:10.3389/fnut.2025.1661993)
Supplement: Supplementary file 6 [file Table_5.docx]

**Supplementary Table 5**. Association between PNI and NRM in patients with aGVHD following allo-HSCT

| Characteristic | Total,  n (%) | Event,  n (%) | Crude model | |  | Model 1 | |  | Model 2 | |  | Model 3 | |
| --- | --- | --- | --- | --- | --- | --- | --- | --- | --- | --- | --- | --- | --- |
|  |  |  | HR (95%CI) | *p*-Value |  | HR (95%CI) | *p*-Value |  | HR (95%CI) | *p*-Value |  | HR (95%CI) | *p*-Value |
| OS |  |  |  |  |  |  |  |  |  |  |  |  |  |
| PNI | 109 | 66 (60.6) | 0.95 (0.92~0.98) | 0.003 |  | 0.94 (0.91~0.98) | 0.004 |  | 0.93 (0.91~0.97) | <0.001 |  | 0.95 (0.90~0.99) | 0.019 |
| PNI category |  |  |  |  |  |  |  |  |  |  |  |  |  |
| T1 | 36 | 25 (69.4) | 1(Ref) |  |  | 1(Ref) |  |  | 1(Ref) |  |  | 1(Ref) |  |
| T2 | 36 | 20 (55.6) | 0.61 (0.34~1.10) | 0.101 |  | 0.62 (0.35~1.12) | 0.102 |  | 0.38 (0.18~0.78) | 0.008 |  | 0.41 (0.19~0.88) | 0.023 |
| T3 | 37 | 21 (56.8) | 0.44 (0.24~0.79) | 0.007 |  | 0.44 (0.23~0.81) | 0.009 |  | 0.23 (0.11~0.5) | <0.001 |  | 0.34 (0.14~0.82) | 0.017 |
| Trend test |  |  |  | 0.007 |  |  | 0.009 |  |  | <0.001 |  |  | 0.015 |

PNI, Prognostic Nutritional Index; NRM,Non-relapse mortality;T1, PNI (20.15-35.8); T2, PNI (35.8-42.5); T3, PNI(42.5-59.7); aGVHD, acute graft-versus-host disease; allo-HSCT, allogeneic hematopoietic stem cell transplantation; HR, Hazard Ratio; CI, Confidence Interval; Ref, reference; OS, overall survival; EFS, event-free survival.

Model 1: Adjusted for Age and Sex;

Model2: Adjusted for Model1 and Indication for HSCT, Stem cell sources, Type of transplantation, Conditioning regimen, Days from transplantation to diagnosis, ABO match, MNC count, CD34^+^ cells count;

Model3: Adjusted for Model2 and Granulocyte implantation time, CMV viremia, EBV viremia, White blood cells, Hemoglobin and Platelets, Total bilirubin, Creatinine, Pulmonary infection, Intestinal infection, Febrile neutropenia, aGVHD grade.
